# Response Across the Health-Literacy Spectrum of Kidney Transplant Recipients to a Sun-Protection Education Program Delivered on Tablet Computers: Randomized Controlled Trial

**DOI:** 10.2196/cancer.4787

**Published:** 2015-08-18

**Authors:** June K Robinson, John J Friedewald, Amishi Desai, Elisa J Gordon

**Affiliations:** ^1^ Department of Dermatology Northwestern University Feinberg School of Medicine Chicago, IL United States; ^2^ Comprehensive Transplant Center Northwestern University Feinberg School of Medicine Chicago, IL United States; ^3^ Division of Nephrology, Department of Medicine University of Illinois at Chicago Chicago, IL United States; ^4^ Center for Healthcare Studies Northwestern University Feinberg School of Medicine Chicago, IL United States

**Keywords:** culturally sensitive, electronic health intervention, kidney transplant recipients, post-transplant outcomes, skin cancer, squamous cell carcinoma, sun protection, tablet computer, patient education, mobile health

## Abstract

**Background:**

Sun protection can reduce skin cancer development in kidney transplant recipients, who have a greater risk of developing squamous cell carcinoma than the general population.

**Objective:**

A culturally sensitive sun-protection program (SunProtect) was created in English and Spanish with the option of choosing audio narration provided by the tablet computer (Samsung Galaxy Tab 2 10.1). The intervention, which showed skin cancer on patients with various skin tones, explained the following scenarios: skin cancer risk, the ability of sun protection to reduce this risk, as well as offered sun-protection choices. The length of the intervention was limited to the time usually spent waiting during a visit to the nephrologist.

**Methods:**

The development of this culturally sensitive, electronic, interactive sun-protection educational program, SunProtect, was guided by the “transtheoretical model,” which focuses on decision making influenced by perceptions of personal risk or vulnerability to a health threat, importance (severity) of the disease, and benefit of sun-protection behavior. Transportation theory, which holds that narratives can have uniquely persuasive effects in overcoming preconceived beliefs and cognitive biases because people transported into a narrative world will alter their beliefs based on information, claims, or events depicted, guided the use of testimonials. Participant tablet use was self-directed. Self-reported responses to surveys were entered into the database through the tablet. Usability was tested through interviews. A randomized controlled pilot trial with 170 kidney transplant recipients was conducted, where the educational program (SunProtect) was delivered through a touch-screen tablet to 84 participants.

**Results:**

The study involved 62 non-Hispanic white, 60 non-Hispanic black, and 48 Hispanic/Latino kidney transplant recipients. The demographic survey data showed no significant mean differences between the intervention and control groups in age, sex, income, or time since transplantation. The mean duration of program use varied by the ethnic/racial group, with non-Hispanic whites having the shortest use (23 minutes) and Hispanic/Latinos having the longest use (42 minutes). Knowledge, awareness of skin cancer risk, willingness to change sun protection, and use of sun protection increased from baseline to 2 weeks after the program in participants from all ethnic/racial groups in comparison with controls (*P*<.05). Kidney transplant recipients with inadequate (47/170, 28%) and marginal functional health literacy (59/170, 35%) listened to either Spanish or English audio narration accompanying the text and graphics. After completion of the program, Hispanic/Latino patients with initially inadequate health literacy increased their knowledge more than non-Hispanic white and black patients with adequate health literacy (*P*<.05). Sun protection implemented 2 weeks after education varied by the ethnic/racial group. Outdoor activities were reduced by Hispanics/Latinos, non-Hispanic blacks sought shade, Hispanic/Latinos and non-Hispanic blacks wore clothing, and non-Hispanic whites wore sunscreen (*P*<.05).

**Conclusion:**

Educational program with a tablet computer during the kidney transplant recipients’ 6- or 12-month follow-up visits to the transplant nephrologist improved sun protection in all racial/ethnic groups. Tablets may be used to provide patient education and reduce the physician’s burden of educating and training patients.

**Trial Registration:**

ClinicalTrials.gov NCT01646099; https://clinicaltrials.gov/ct2/show/NCT01646099

## Introduction

### Background

Sun protection is important for kidney transplant recipients, as they have a 20- to 100-time greater risk of developing squamous cell carcinoma (SCC) than the general population [1]. Effective sun protection has been reported to reduce the development of SCC in non-Hispanic white kidney transplant recipients when practiced over a 2-year period [2]. The impaired quality of life experienced by kidney transplant recipients from disfigurement and loss of function from many surgical procedures to remove SCC, as well as the anxiety and fear about the return or spread of SCC, may be alleviated by implementing sun protection, which would reduce the risk of developing SCC.

Although non-Hispanic white kidney transplant recipients with skin that sunburns easily and tans poorly have the greatest risk of developing SCC, SCC also occurs in many Hispanic/Latinos and non-Hispanic blacks [3]. Hispanic/Latinos and non-Hispanic blacks display considerable diversity in the sun sensitivity of their skin. Some individuals in these groups have sun-sensitive skin [4]. The term “people with skin of color,” bridges descriptions of race and ethnicity, and allows self-identification by those with mixed ethnicity/race. People with skin of color commonly do not perceive sunburn/skin cancer as relevant because they and their families lack sufficient experience with sunburn/skin cancer as well as with using sun protection [5]. Because of increased skin cancer risk, effective sun-protection counseling is needed for all kidney transplant recipients, regardless of skin color. However, the need for culturally sensitive sun-protection counseling of kidney transplant recipients with skin of color may be unrecognized by patients and providers due to the assumption that their skin color provides sufficient sun protection.

The development of this culturally sensitive, electronic, interactive sun-protection educational program, SunProtect, was guided by the transtheoretical model, which focuses on decision making influenced by perceptions of personal risk or vulnerability to a health threat, importance (severity) of the disease, and benefit of a behavior to the health outcome [6]. Because the tablet personal computer (tablet) supports presentation of videos, we created storytelling testimonials in English and Spanish with non-Hispanic white, Hispanic Latino, and non-Hispanic black kidney transplant recipients. Our decision to develop video testimonials was guided by transportation theory, which holds that narratives can have uniquely persuasive effects in overcoming preconceived beliefs and cognitive biases because people transported into a narrative world will alter their beliefs based on information, claims, or events depicted [7,8]. These videos specifically aim to improve knowledge about skin cancer and address relevance to people with skin of color by giving personal details about developing skin cancer and sun-protection use. Patients identify with the storytellers, which increases the likelihood that social influence will shift their normative beliefs.

### Objective

**Figure 1:**
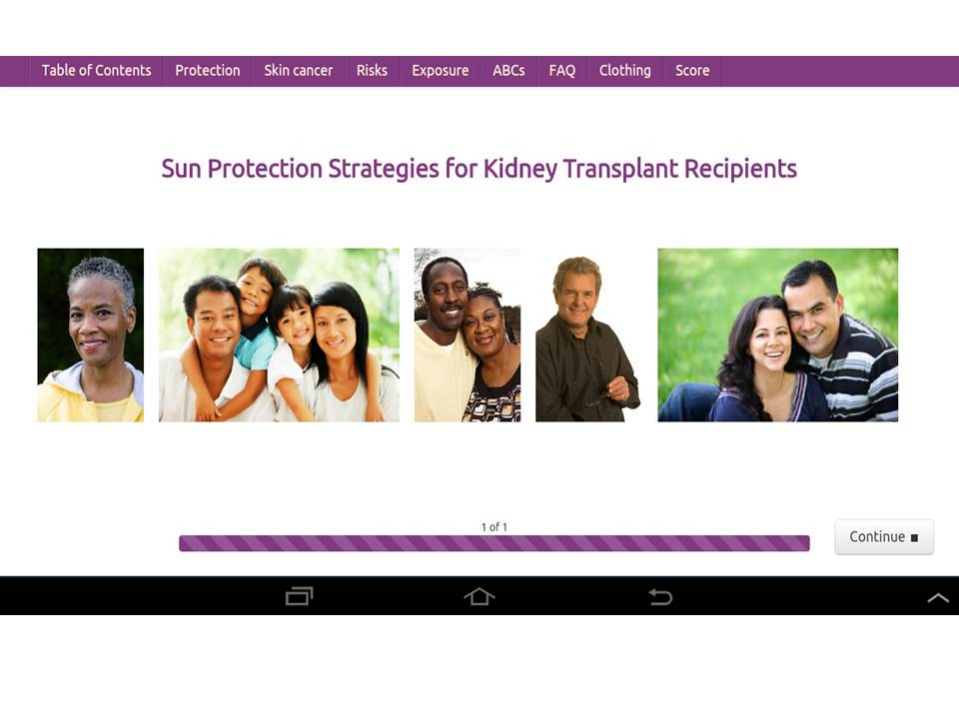
Introduction to the program showed the range of ethnicities and races.

SunProtect was particularly developed for patients with lower health literacy and from racially/ethnically diverse backgrounds, which is especially important given that minorities comprise 42% of living kidney transplant recipients in the United States [9] (Figure 1). In addition, approximately one third of kidney transplant recipients have limited health literacy, and thus, the educational content included terms that are easily understandable through multiple interactive media, including audio, video, pictorial, and textual information written below a 6th-grade reading level [10-12]. This pilot research evaluated the impact of SunProtect on knowledge, intentions to use sun protection, and use of sun protection among non-Hispanic white, Hispanic Latino, and non-Hispanic black kidney transplant recipients before and 2 weeks after education.

## Methods

### Setting and Recruitment

Kidney transplant recipients from 2 urban Chicago programs, Northwestern Medicine and University of Illinois at Chicago, were eligible for participation in the study if they met the following inclusion criteria: (1) had received a kidney transplantation within the past 2-24 months, (2) spoke and read English or Spanish, (3) aged between 18 and 70 years, (4) could see well enough to read a newspaper, (5) lived in the greater Chicago area, and (6) self-identified as non-Hispanic white, non-Hispanic black, or Hispanic Latino. Patients were excluded from the study if they had a previous self-reported history of skin cancer as verified in their medical record, previously participated in sun-protection educational research conducted by this research team, a history of dermatologic disease treated with ultraviolet light (eg, psoriasis, atopic dermatitis), and were under the care of a dermatologist within the last 5 years.

Research coordinators recruited potential participants by calling them (over telephone) 1 week before their scheduled appointment with the transplant nephrologist. Patients were told about the sun-protection educational study and asked if they were interested in participating during their visit to the transplant nephrologist and 2 weeks later by telephone.

### Design

#### Accrual

Accrual was purposefully stratified to obtain representation of all 3 ethnic/racial groups. Written consent was obtained by a research assistant. From mid-May to mid-July 2014, consenting participants used a tablet to complete an online self-report pretest survey in the physicians’ offices. Immediately after completing the pretest, participants were randomized using a random allocation sequence for each ethnic/racial group (1:1) to receive SunProtect, an educational sun-protection program, or to be in the control group. The control group received general skin care information. The software program gave the participants their allocation, and thus, the kidney transplant recipients were not blinded to their condition. Two weeks later, participants were called by a research assistant, who did not enroll the kidney transplant recipient and was blinded to their condition, and asked to respond to the same sun-protection survey questions used in the pretest. The Institutional Review Boards of Northwestern University and the University of Illinois Hospital and Health Sciences System approved the study and participants were compensated for study participation.

#### Educational Sun-Protection Program

SunProtect was derived from an educational sun-protection workbook created and used in our prior research [13]. Sun-protection options described in SunProtect included restricting outdoor exposure between 10 am and 4 pm, seeking shade when outdoors, wearing protective clothing (hats, long sleeved shirts, long pants, and sunglasses), and/or applying broad-spectrum sunscreen with a sun-protection factor of 30 or more.

Text screens (n=36) were evaluated with the Flesch-Kincaid test to assure that the grade level did not exceed 6th-grade reading level. SunProtect had an introductory section in which program navigation was demonstrated, and the language (English or Spanish) and audio guide were selected by touching one of the following 4 images: non-Hispanic white woman, Hispanic Latino woman, Hispanic Latino man, or non-Hispanic black man. The topics of 8 sequential chapters were as follows: importance of sun protection, skin cancer, risk of developing skin cancer, ways people get sun exposure, choices of sun protection, frequently asked questions about sunscreen, protective clothing, and personalized sun-protection recommendations (Table 1). Videos demonstrated effective sunscreen application. Participants used headphones to listen to the program in the waiting room.

**Table 1:** SunProtect content.

Chapter	Screen (N)	Supplemental content	
		Photographs Title (N)	Video and testimonials title (playing time in seconds)
Navigation	1		
Select language + program audio guide	1	5	
Table of contents	1		
Why protect against the sun?	1	Sunburn (2), dark spots (2)	
What is skin cancer?	3	Basal cell carcinomas (2), squamous cell carcinomas (2), melanoma (2)	
What is the chance of a kidney transplant recipient getting skin cancer?	6	Kidney transplant recipient’s risk (1), public’s risk (1), select skin tone (1), ease of sunburn (2), time from transplant to get skin cancer (1)	Testimonial: Surprised by sunburn (10)
How do people get sun exposure?	3	Outdoor activities (2): Select outdoor activities you do	
ABC rule for sun protection	3	*A*void sun (1), *b*lock sun (1), *c*over up in the sun (1)	Testimonials (3), skin irritation from sun (7), using sunblock (9), wearing a hat (8)
Frequently asked questions about sunscreen	9	Broad-spectrum sun blockage (3), amount to use (1), two coats of sunscreen (1)	Lotion, gel, cream, and stick sunscreen consistency (77); application of sunscreen stick (32); sunscreen spray to arm (20); sunscreen lotion (31); difficulty of spraying sunscreen on your own back (14); incorrect sunscreen spraying (10); application of sunscreen lotion to face: correct (35 seconds)/incorrect (21 seconds)
What types of protective clothing are good to wear?	2	Protective clothing (1), correct hat (2)	
Your personal sun-protection recommendation: • Request personal sun-protection recommendation • Request tip for early recognition of skin cancer	6	Doctor gives personal recommendation for sun protection (1), tips to remember sun-protection gear (1)	

**Figure 2:**
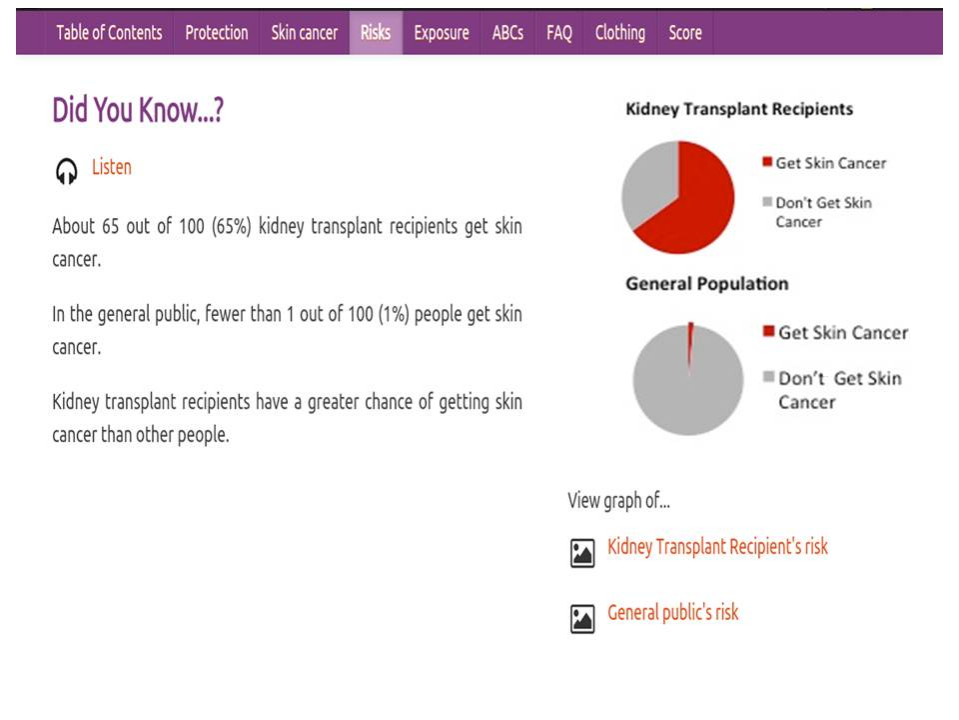
Users toggled between the 2 pie graphs presenting the risk of developing skin cancer in kidney transplant recipients and in the general population.

The tablet screen was divided into 3 parts with the primary content on the left half of the screen. Supplementary content, which was available on the right side of the screen, was accessed by touching the icon (Figure 2). SunProtect was displayed on a Samsung Galaxy Tab 2 10.1, a tablet personal computer (tablet) with a touch screen and was created in collaboration with the Center for Behavioral Intervention Technologies at Northwestern University.

Upon touching the headphone icon, audio narration presenting the same content as the written text was available for all program screens. Users could elect not to listen to the audio narration, skip pages and chapters, or repeat them.

#### Personalizing the Sun-Protection Recommendation

**Figure 3:**
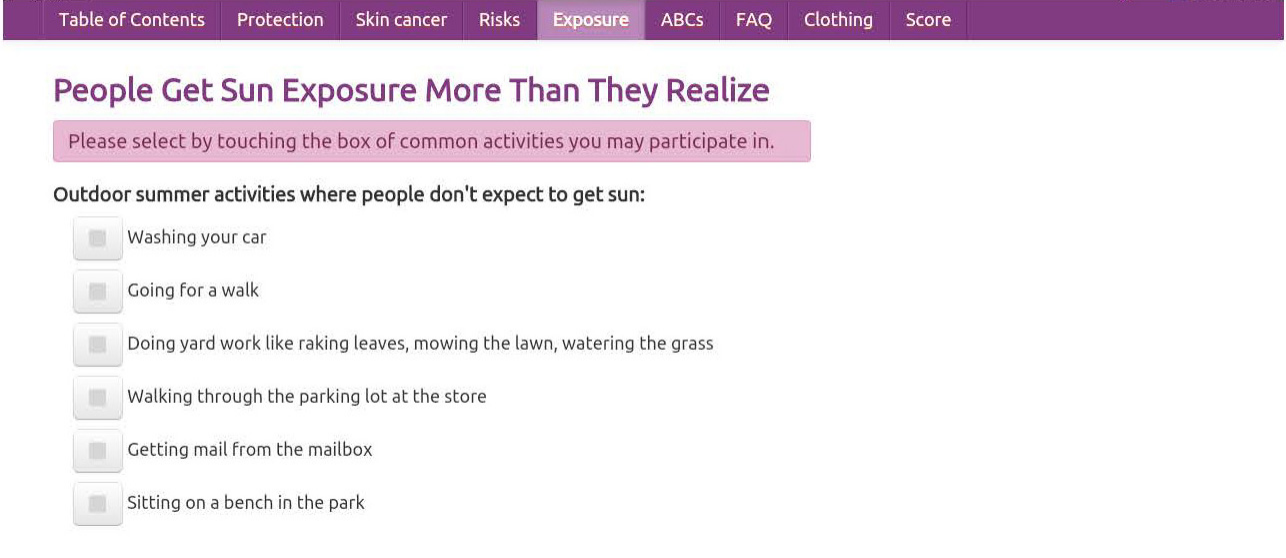
Users selected daily activities with commonly unrecognized sun exposure.

**Figure 4:**
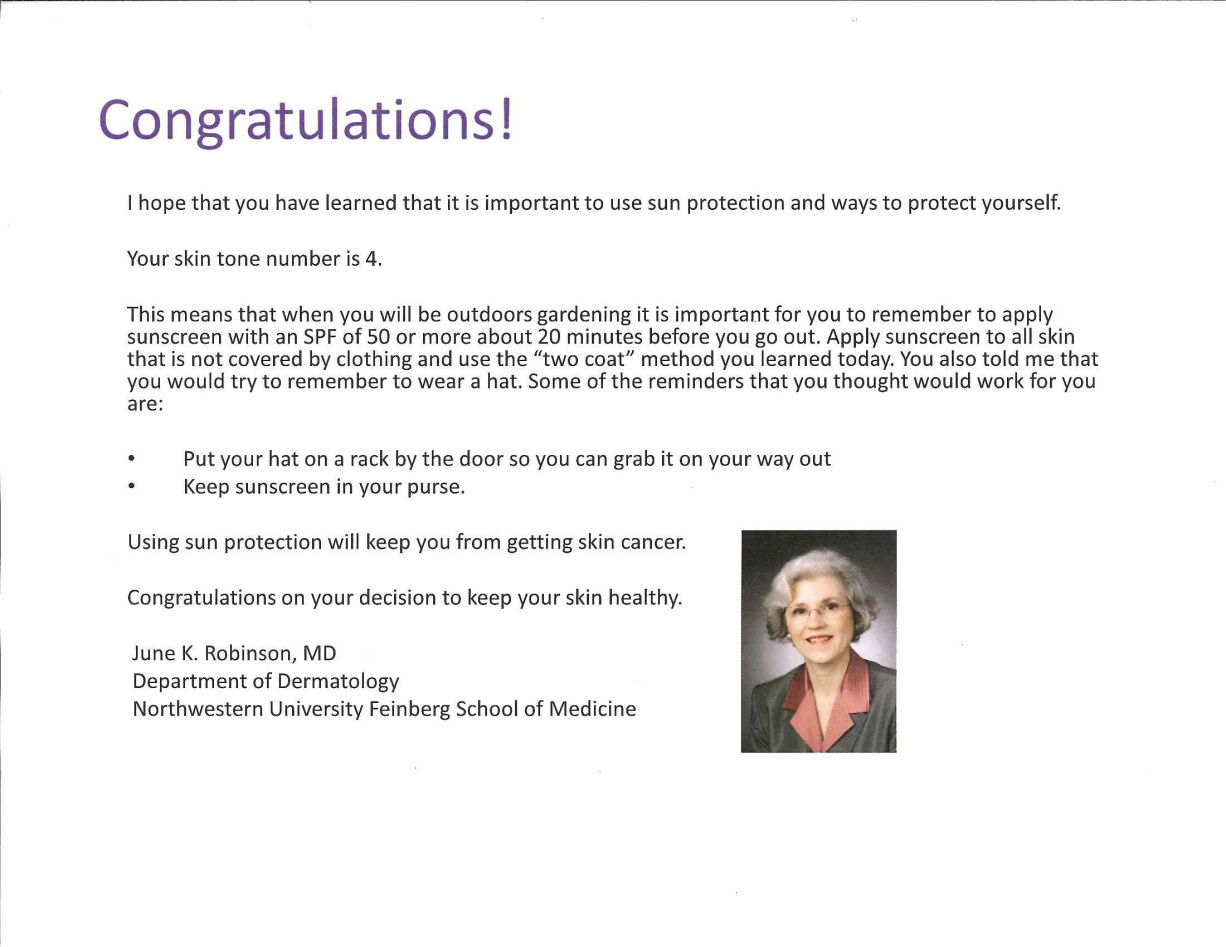
Personalized sun-protection recommendation from the doctor.

The educational tablet program included interactive components in which the participants selected (1) the color bar that most closely matched the color of their skin in the sun-protected location of the upper inner arm, (2) the daily outdoor activities they usually performed, and (3) their commonly used sun-protective behaviors, if any (Figure 3). These 3 self-reported items were used to develop tailored sun-protection recommendations for each user. Their personal sun-protection recommendations were delivered at the end of the SunProtect program by a physician appearing on screen (Figure 4).

#### Cultural Sensitivity

At the beginning of the program, participants selected the preferred language, English or Spanish, and their choice of narrators. Two Hispanic Latino bicultural, bilingual research coordinators translated the text and audio narration of the program from English to Spanish. Several bilingual physicians and health professionals then validated the translation of the text and audio narration from Spanish to English.

Culturally appropriate language (eg, the term “skin irritation” from the sun) was used in place of “sunburn” to describe the response of people with skin of color to sun exposure. Sunburn and skin irritation from sun exposure were depicted as occurring in people with skin of color as well as in the skin of non-Hispanic white people after “getting some sun.” Language such as “tan” as a response to sun exposure was explicitly avoided because in our previous research, non-Hispanic blacks and Hispanic Latinos reported that they “got dark” rather than “tan” [5].

Because many non-Hispanic black kidney transplant recipients did not know how to swim, examples of outdoor activities commonly enjoyed by non-Hispanic white, such as swimming, were not used in the workbook. Family outdoor activities were emphasized because Hispanic Latino kidney transplant recipients noted the importance of time spent with the extended family (eg, a picnic in the park).

**Figure 5:**
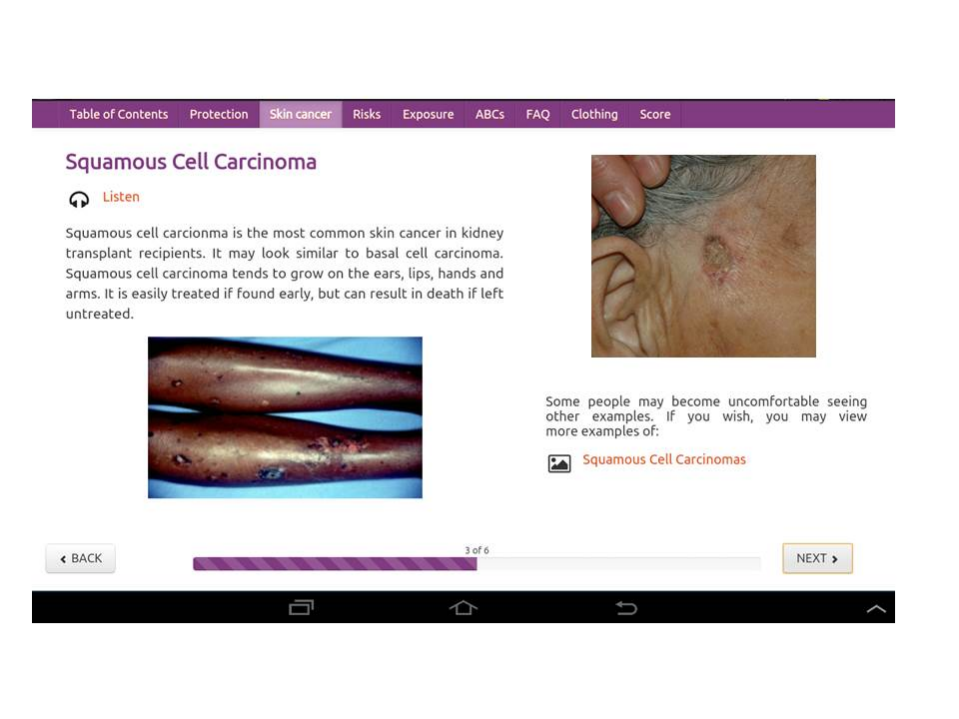
Explanation of squamous cell carcinoma with examples in kidney transplant recipients with skin of color.

**Figure 6:**
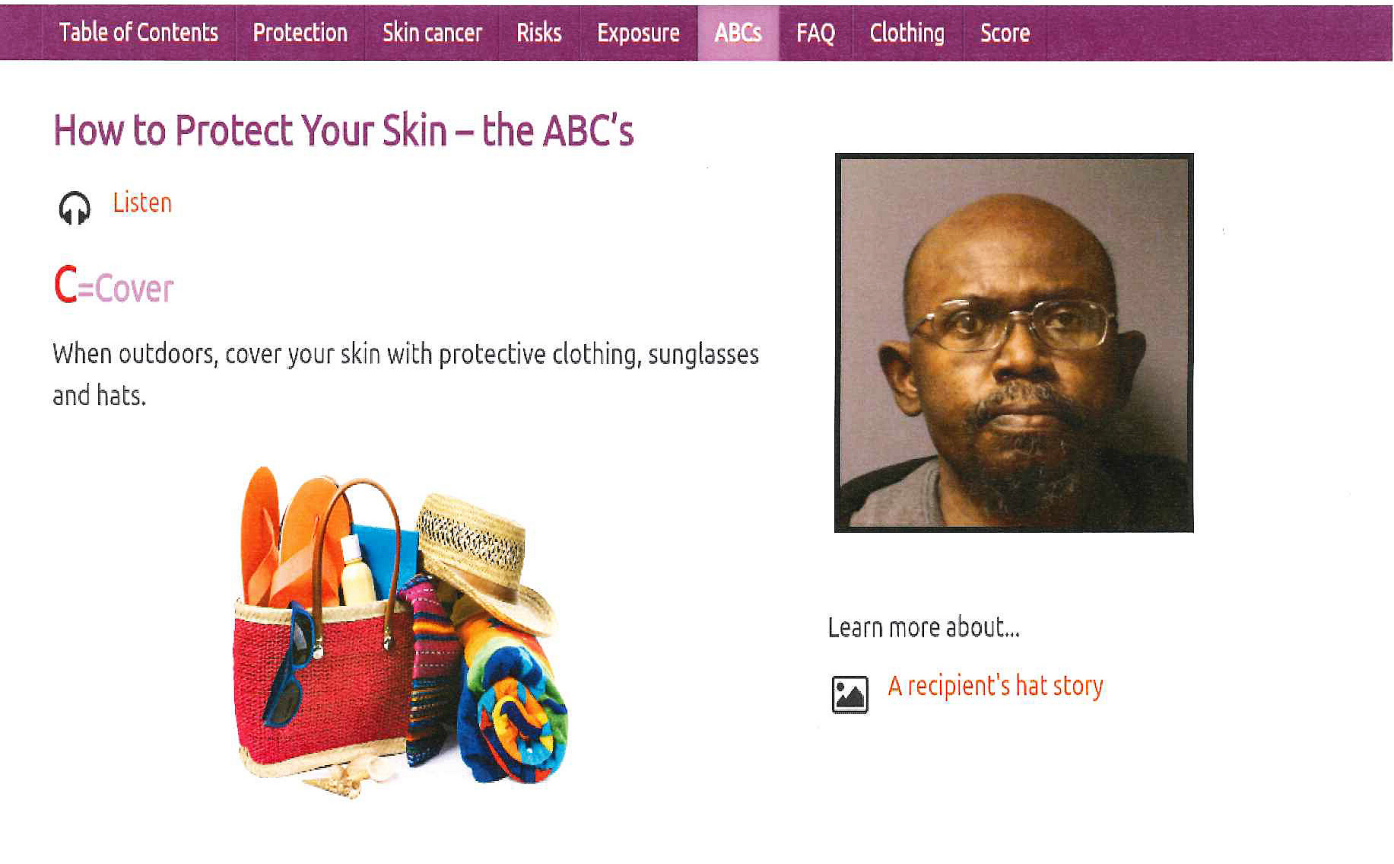
Testimonial from a non-Hispanic black kidney transplant recipient relating skin irritation from the sun on his bald scalp because he forgot his hat.

Lastly, photographs of skin cancers and skin changes from sun exposure occurring in people with all skin tones were presented (Figure 5). The audio narration of images of sunburn and skin irritation addressed non-Hispanic white, non-Hispanic black, and Hispanic Latino people’s beliefs about preferences for and the ability to get darker skin color, skin irritation, and skin cancer from exposure to the sun. For example, the audio accompanying a picture of a non-Hispanic black man with a bit of pink color on his cheek stated, “People with skin of color that in the past may not have gotten pink from being out in the sun may get a bit pink after their transplant.” The picture of a Latina with dark spots on her face was accompanied by the following narration: “Many Latinas use sun protection to keep from getting dark spots from the sun.” A bald non-Hispanic black man, who was a kidney transplant recipient, related his story of getting a sun irritation on his bald head when he went to Florida to Disney World with his grandchildren on spring break and forgot to take his hat (Figure 6). The content did not change during the randomized controlled trial (Trial Registration: ClinicalTrials.gov NCT01646099).

### Measures

#### Health Literacy

Health literacy was ascertained by a written self-administered survey in Spanish or English of the Short Test of Functional Health Literacy in Adults (S-TOFHLA) administered as a timed 7-minute 36-item survey [14]. Health literacy was categorized into one of the following 3 groups: adequate health literacy (36-23), marginal health literacy (22-17), and inadequate health literacy (16-0).

#### Demographic Information, Knowledge, Attitudes, and Sun-Protection Behavior

A brief self-report survey of demographic information was administered on the tablet. Participants could select the English or Spanish versions to read and could elect to hear the audio narration. People with inadequate health literacy as determined by the score received on the S-TOFHLA were invited to have the survey administered by the research coordinator. Demographic information consisting of sex, age, marital status, race/ethnicity, education, annual household income, and months since receiving the transplant was only assessed in the first survey.

Knowledge of skin cancer and sun protection was assessed with the following 9 statements with “agree” or “disagree” responses: (1) people with a kidney transplant take medicine that may make their skin sensitive to the sun; (2) only people with a kidney transplant who have sun-sensitive skin, who freckle and sunburn easily, have to worry about getting a skin cancer; (3) applying sunscreen after being out in the sun is enough protection; (4) when the sun is high in the sky, seek shade to avoid the strong rays of the sun; (5) clothing does not protect the skin from the sun; (6) sunglasses protect the delicate skin around the eyes; (7) it takes about a teaspoon of sunscreen to cover the skin of the whole body; (8) a baseball cap is a good hat for sun protection; (9) walking outside at noon for an hour is not enough time outside to need sun-protective gear. Recognition of their personal risk of developing skin cancer was an item with a 5-point Likert scale. Hours of outdoor sun exposure were assessed by their weekday and weekend duration of outdoor activities between 10 am and 4 pm. Willingness to change sun-protection behavior (20 items with a 5-point Likert scale) and current sun-protection behavior(s) focused on the use of sun protection by wearing sunscreen, wearing protective clothing, wearing sunglasses, and seeking shade (20 items with a 5-point Likert scale). The same items were assessed with all participants by a self-report survey at 2 study time points (before and 2 weeks after the baseline visit). The psychometrically validated measures among kidney transplant recipients were previously reported [13]. The survey items, which could be “read” to the kidney transplant recipients selecting the audio version, elicited the same responses online or in writing in a sample of 20 kidney transplant recipients.

#### Educational Program Evaluation: Usability, Use, and Satisfaction

To test the “usability” of SunProtect, research coordinators performed 1-2-hour cognitive interviews with 4 non-Hispanic white and 4 non-Hispanic black kidney transplant recipients in English, and 5 Hispanic Latino kidney transplant recipients in Spanish, which were audio and video recorded. Each interview was transcribed and translated into English to (1) evaluate and provide feedback on the overall look of the screen, the font size, color scheme, and navigation buttons; (2) solicit suggestions for improving behavioral alternatives; and (3) evaluate the cultural sensitivity of the tablet program. Three reviewers independently reviewed the audiotapes, field notes, and coded the data. The team met to discuss the interpretations, come to consensus, and identify data-driven approaches to revise the content. Revisions to content presented on the screens of the tablet were made in an iterative process after conducting 3 interviews with at least one individual from each racial/ethnic group.

Measures of the “use” of SunProtect were the duration of program use, the time spent on an individual page/screen, repetition of any portion of a chapter, selection of audio narration, and viewing of supplementary figures, videos, and/or testimonials.

Participants rated their “satisfaction” with the program’s ease of use, usefulness of content, and visual appeal of the presentation on a 5-point scale, with 5 being the greatest ease of use, content usefulness, and appeal of presentation.

#### Written Material Requested

At the end of the program, participants had the option to request that their personal sun-protection recommendations and/or the tip sheet for early detection of SCC with color illustrations be sent by email [15].

#### Participant Waiting Time

Research coordinators observed and kept a log of the time kidney transplant recipients spent in the waiting room until they were seen by the transplant nephrologists.

#### Statistical Analysis and Sample Size

The sample size required to sensitively detect a 30% difference in using sun protection between the 3 ethnic/racial groups was 180 (60 in each group completing the study), assuming an alpha less than .05 and power of 0.8 or more in a two-tailed test. Attrition was estimated at 20%, with N=60 in each group. The effect size in each group was 20 with 95% confidence interval (±1-39).

Program use and evaluation, change in knowledge, and requests for written materials were compared between groups using Chi-square tests of association and Wilcoxon rank sum tests. Summary statistics are presented as counts and percentages, or mean (standard deviation) as appropriate. All analyses were run at a nominal type I error rate of 5%, and performed in SAS version 9.2 (Cary, NC, USA).

## Results

### Population

Of the 522 eligible kidney transplant recipients approached, 170 were accrued to the study (170/522, 32.6% participation rate). Eight eligible recipients at the University of Illinois at Chicago could not enter the study due to failure of the Wi-Fi service. All participants in the baseline assessments completed the 2-week follow-up, including 60 non-Hispanic black, 62 non-Hispanic white, and 48 Hispanic Latino, who were mostly men (101/170, 59.4%) with a mean age of 51 (Table 2). There were no statistically significant differences in race/ethnicity, sex, annual household income, age, time since transplantation, or history of work-related sun exposure between the intervention and control groups. Spanish narration was preferred by Hispanic Latino kidney transplant recipients (45/48, 94%). Twenty-eight percent of the kidney transplant recipients had inadequate health literacy. Inadequate health literacy was present in Hispanic Latino kidney transplant recipients (45/48, 94%; *P*<.05). Non-Hispanic black kidney transplant recipients (45/60, 75%) had marginal health literacy in comparison with non-Hispanic whites (*P*<.05).

**Table 2:** Demographics of population (N=170).

Characteristics		Intervention^a,b^	Standard of care^a,c^	*P* value^d^
**Race/Ethnicity**				.183
	Non-Hispanic white	32 (38.1)	30 (34.9)	
	Hispanic/Latino	23 (27.4)	25 (29.1)	
	Non-Hispanic black	29 (34.5)	31(36.0)	
Male		45 (53.6)	56 (65.1)	.376
Married		49 (58.3)	41 (47.7)	.060
College education or higher		37 (44.0)	30 (34.9)	.099
**Annual household income**				.648
	<10,000	16 (19.0)	14 (16.3)	
	10,000-19,999	12 (14.3)	13 (15.1)	
	20,000-34,999	14 (16.7)	13 (15.1)	
	35,000-50,999	18 (21.4)	14 (16.3)	
	51,000-100,000	13 (15.5)	22 (25.6)	
	>100,000	11 (13.1)	10 (11.6)	
Age in years, mean (SD)		51.0 (12.5)	49.0 (14.2)	.334
Months since transplant, mean (SD)		17.3 (15.1)	18.0 (15.3)	.733
Work-related sun exposure		35 (41.7)	38 (44.2)	.896

^a^Values in the “Intervention” and “Standard of care” columns are provided as n (%), unless indicated otherwise.

^b^N=84

^c^N=86

^d^ *P* values from Chi-square tests of association or t tests

### Participant’s Self-Reported Knowledge, Attitudes, and Sun Protection

Overall, there were significant gains in knowledge, perception of being at risk to develop skin cancer, and willingness to change sun protection for all kidney transplant recipients using the intervention in comparison with controls (Table 3). The increase in knowledge of Hispanic Latino kidney transplant recipients was significantly greater than the increase in knowledge by non-Hispanic white and non-Hispanic black kidney transplant recipients (*P*<.05; Table 3). The greatest willingness to change sun protection was demonstrated by Hispanic Latino kidney transplant recipients (*P*<.05). Sun protection varied by the ethnic/racial group (eg, Hispanic Latino kidney transplant recipients choose to wear clothing, non-Hispanic black kidney transplant recipients seeking shade and wearing clothing, and non-Hispanic whites using sunscreen). Non-Hispanic black kidney transplant recipients, who had markedly fewer daily hours of outdoor exposure than the other 2 groups, did not demonstrate a significant reduction in outdoor exposure (*P*<.05).

**Table 3:** Change in knowledge, intentions to use sun protection, and sun-protection use by kidney transplant recipients.

Participant self-reported variable^a^			Non-Hispanic white N=62			Hispanic/Latino N=48			Non-Hispanic black N=60		Wilcoxon rank sum *P* values
			Intervention N=32		Control N=30	Intervention N=23	Control N=25	Intervention N=29		Control N=31	
**Knowledge (1-10 scale)**												
		Pretest	3 (2.5)		3 (2.1)	2 (0.2)	2 (0.7)	2 (1.8)		2 (1.1)	.04^b^
		Post-test	5 (3.4)		3 (2.5)	8 (1.1)	4 (1.3)	4 (2.6)		5 (2.3)	
**Attitudes**											
	**Recognize personal skin cancer risk (1-5 scale)**												
		Pretest	2 (1.3)		2 (0.7)	1 (0.5)	1 (0.7)	1 (0.6)		1 (0.8)	.02^b^
		Post-test	3 (1.6)		2 (1.1)	4 (0.7)	2 (1.1)	2 (1.7)		1 (0.4)	
	**Willingness to change sun protection (20-100 scale)**												
		Pretest	22 (0.7)		20 (1.0)	22 (1.0)	21 (1.3)	21 (1.0)		22 (1.5)	.01^b^
		Post-test(immediate)	66 (24.3)		21 (0.6)	85 (10.2)	22 (09)	43 (20.2)		23 (2.4)	
**Sun protection used at 2 weeks**											
	**Sun protection (20-100 scale)**												
		Pretest	47 (10.1)		48 (9.7)	28 (4.7)	29 (5.4)	31 (6.9)		30 (4.7)	.01^b^
		Post-test (2 weeks)	60 (11.6)		50 (10.1)	55 (3.9)	30 (1.4)	46 (10.3)		31 (6.3)	
	**Daily hours outdoors (0.5-6 hours)**												
		Pretest	2.4 (0.7)		2.1 (1.4)	3.7 (1.2)	4.0 (2.1)	1.1 (0.5)		1.0 (0.9)	.01^b^
		Post-test (2 weeks)	1.6 (0.9)		2.5 (0.7)	2.4 (1.8)	4.3 (1.9)	1.0 (0.5)		1.0 (0.8)	

^a^All values are reported as mean (SD)

^b^Statistically significant Wilcoxon rank sum test

Participant Concern

Two kidney transplant recipients became concerned about lesions and asked to have them checked by one of the authors (JKR). However, both were benign.

### Participant Waiting Time

The patient waited in the waiting room for about 30 minutes (SD 12 minutes).

### Usability of, Use of, and Satisfaction With the Program

*Usability* testing was performed until no further changes were suggested by the last 3 participants (1 non-Hispanic white, 1 Hispanic Latino, and 1 non-Hispanic black). The button and font sizes were changed, and the icon symbols were enlarged.

The mean duration of “use” was 27 minutes (range 23-42 minutes) with Hispanic Latino kidney transplant recipients using the program the longest (Table 4). Compared with non-Hispanic white kidney transplant recipients, Hispanic Latino and non-Hispanic black kidney transplant recipients spent significantly longer time viewing “why protect against the sun” and repeatedly viewed the images of sunburn occurring in people with all types of skin (*P*<.05). Hispanic/Latino and non-Hispanic black kidney transplant recipients also spent a significantly longer time viewing the images of skin cancer and the videos about sunscreen types and application methods than did non-Hispanic white kidney transplant recipients (*P*<.05).

**Table 4:** Use and evaluation of SunProtect by kidney transplant recipients.

Variable		Non-Hispanic white^a^	Hispanic/Latino^b^	Non-Hispanic black^c^	Wilcoxon rank sum *P* value
**Program use**					
	Mean duration (minutes)	23	42	32	.046^d^
**Screens repeated** ^e^					
	Why protect against the sun	1 (0.2)	1 (3.4)	1 (2.7)	.02^d^
	What is skin cancer	3 (4.1)	7 (5.6)	5 (4.9)	.03^d^
	Chance of getting skin cancer	2 (4.1)	2 (4.6)	2 (4.3)	.45
	Sunscreen application	1 (5.0)	4 (9.1)	3 (8.2)	.03^d^
**Program evaluation** ^f^					
	Ease of use	4 (0.5)	4 (0.5)	4 (0.2)	.85
	Usefulness of content	5 (0.0)	5 (0.0)	4 (0.6)	.70
	Appealing presentation	4 (1.0)	4 (0.7)	4 (1.0)	.73
**Request written material** ^g^					
	Sun-protection recommendations	12/32 (37.5)	10/23 (43.5)	9/29 (31.0)	.64
	Tips to detect skin cancer	27/32 (84.4)	19/23 (82.6)	24/29 (82.8)	.85

^a^N=32

^b^N=23

^c^N=29

^d^Statistically significant Wilcoxon rank sum test

^e^Values presented as number of times repeated (SD)

^f^Values presented as mean (SD), on a scale of 1-5

^g^Values presented as n/N (%)

There was no significant difference among the 3 racial/ethnic groups in the duration of viewing the section on risk of developing skin cancer (4.1 minutes by non-Hispanic white, 4.6 by Hispanic Latino, and 4.3 by non-Hispanic black) or the number of repetitions of screens. On average, users “toggled” between the pie charts depicting relative risk rates for developing skin cancer for kidney transplant recipients and the general population 4 times (Figure 1).

Patients without prior experience using a tablet were not able to implement the navigation directions presented on the tablet (45 Hispanic Latinos, 16 non-Hispanic blacks, 5 non-Hispanic whites, 66/170, 39%). For users without prior experience using a tablet, the research assistant demonstrated screen navigation for 39% of users and stayed in an adjacent room to be available to assist the participant with concerns. Seven participants required further assistance in accessing the videos.

Spanish “audio narration” was chosen by Hispanic Latino kidney transplant recipients with inadequate health literacy (45/48). Most non-Hispanic white kidney transplant recipients, who had adequate health literacy, did not choose the audio narration (48/60). Inadequate and marginally functional health literate kidney transplant recipients chose the audio narration in either Spanish or English with statistically significantly greater frequency than kidney transplant recipients with adequate functional health literacy (*P*<.05).

Participants from all racial/ethnic groups were “satisfied” with the ease of use of the program (4/5), and with the visual appeal of the presentation of content (4/5). The content was deemed useful (5/5) by non-Hispanic white and Hispanic Latino kidney transplant recipients; however, non-Hispanic black kidney transplant recipients felt the content was less useful (4/5).

### Requests for Written Material

While the requests for personal sun-protection recommendations were limited to 20% or less of the participants, more than 80% of participants requested the tip sheet for early detection of SCC with color illustrations.

## Discussion

### Principal Findings

The results of this pilot study found that SunProtect, as delivered by tablet, was effective in increasing knowledge, perception of being at risk of developing develop skin cancer, willingness to change sun protection, and change in sun protection 2 weeks after education among kidney transplant recipients with a range of health literacy. Hispanic/Latino kidney transplant recipients had the least health literacy and gained the greatest increase in knowledge and willingness to change. Participants appeared to have benefited from the ability to listen to the audio presentation in the language of their choice, to move at their own pace, and to repeat screens and chapters to inform their sun-protection decisions. In this research, the 3 most commonly repeated content areas were kidney transplant recipients with skin of color getting sunburn or skin irritation from the sun, how to use sunscreen, and explanations of skin cancer. The chapter about skin cancers had the longest duration of use and the most repetition of content. Knowledge gained by the kidney transplant recipients with inadequate literacy, who elected to listen to the audio descriptions of skin cancer photographs, was greater than by the kidney transplant recipients with adequate literacy, who chose to read the narrative descriptions and did not listen to the audio description. The most frequently requested written material was the tips for early detection of skin cancer. While lack of prior experience using a tablet may have contributed to longer duration of use by some participants, the interest in learning about skin cancer as demonstrated by length of viewing and number of repetitions was common to all participants.

SunProtect provided health information in a manner that was well suited and liked by patients with limited health literacy. Patients with limited health literacy may be less likely to ask questions than others [16]. By listening to the program in their choice of language and repeatedly viewing the images, kidney transplant recipients with limited health literacy were able to learn. The program informed, taught, and counseled using examples of skin cancers occurring on kidney transplant recipients with skin of color, provided testimonials and videos intended to help the kidney transplant recipient understand, reach a decision, and make a choice about sun protection. The tablet format was an effective medium of health communication because it was uniquely able to provide video content and testimonials that could not be made available in face-to-face communication with the health care provider. Written materials do not provide the same level of engagement for the user as the tablet can provide by allowing kidney transplant recipients to choose to listen to the testimonial of a patient. For example, self-directed patients used the tablet to repeatedly view and compare the pie graphs communicating their risk of developing skin cancer. Previous studies have demonstrated greater adherence with sunscreen use among participants educated with videos than those educated with pamphlets [17,18].

Health care providers have limited amounts of time to spend with each patient. The material presented by the tablet supplements customary education provided by health care providers. Furthermore, the usual waiting time for kidney transplant recipients was approximately 30 minutes, which will allow the tablet to be used in the waiting room before the meeting their health care providers. Lastly, when provider and patient do not share a common language, attempts to bridge the language barrier may be difficult; thus, the Spanish language provided by the tablet in text on the screen with audio narration and in the videos may help communicate the sun-protection options.

### Limitations

A limitation of this study is the 2-week period of follow-up. A longer follow-up would be needed to determine whether there was decay in sun-protection behavior. The research was performed in a city with temperate weather, and thus, it may not be possible to generalize the findings to locations with longer periods of sunny weather. In the interest of decreasing participants’ fatigue, confidence in their ability to perform sun protection was not determined. An additional limitation was obtaining the self-reported outcome measures in 2 different ways. The pretest survey was completed on the tablet by the participant. Two weeks after the education, the survey responses were obtained by telephone interview, which may have introduced observer effect. Furthermore, enrollment of Hispanic Latino kidney transplant recipients did not achieve the sample size of 60 completing the study that was needed for analysis of ethnic/racial differences in adoption of sun-protection behavior(s).

### Conclusions

Kidney transplant recipients from diverse racial/ethnic groups and health literacy levels who used SunProtect became aware of their risk of developing skin cancer, increased their knowledge of skin cancer and sun protection, showed willingness to change their sun protection, and changed their sun-protection behavior. Because presurvey and postsurvey items would not be used in routine practice, reinforcement of the need for and relevance of sun protection by the survey will not be done. Regular life-long sun protection can decrease kidney transplant recipients’ chance of developing skin cancer. Future research is needed to examine kidney transplant recipient’s long-term adherence to sun protection and feasibility of delivery in a variety of practices.

Delivery of education with a tablet equipped with headphones may be done in the waiting room before a regularly scheduled 6- or 12-month follow-up visits to the transplant nephrologist. Technology may be used to provide patient education and reduce the physician’s burden of educating and training patients.

## Abbreviations

SCC: squamous cell carcinoma
S-TOFHLA: Short Test of Functional Health Literacy in Adults

## References

[1] Euvrard S, Kanitakis J, Claudy A (2003). Skin cancers after organ transplantation. N Engl J Med.

[2] Ulrich C, Jürgensen JS, Degen A, Hackethal M, Ulrich M, Patel MJ, Eberle J, Terhorst D, Sterry W, Stockfleth E (2009). Prevention of non-melanoma skin cancer in organ transplant patients by regular use of a sunscreen: A 24 months, prospective, case-control study. Br J Dermatol.

[3] Buoy AG, Yoo S, Alam M, Ortiz S, West DP, Gordon EJ, Robinson JK (2010). Distribution of skin type and skin cancer in organ transplant recipients. Arch Dermatol.

[4] Galindo GR, Mayer JA, Slymen D, Almaguer DD, Clapp E, Pichon LC, Hoerster K, Elder JP (2007). Sun sensitivity in 5 US ethnoracial groups. Cutis.

[5] Robinson JK, Joshi KM, Ortiz S, Kundu RV (2011). Melanoma knowledge, perception, and awareness in ethnic minorities in Chicago: Recommendations regarding education. Psychooncology.

[6] Prochaska JO, Norcross JC, Diclemente CC (1994). Changing for Good: The Revolutionary Program That Explains the Six Stages of Change and Teaches You How to Free Yourself From Bad Habits.

[7] Green MC, Brock TC (2000). The role of transportation in the persuasiveness of public narratives. J Pers Soc Psychol.

[8] Green M (2006). Narratives and cancer communication. J Commun.

[9] US Organ Procurement and Transplantation Network and the Scientific Registry of Transplant Recipients (2012). 2012 Annual Report of the US Organ Procurement and Transplantation Network and the Scientific Registry of Transplant Recipients: Transplant Data.

[10] Chisholm MA, Fair J, Spivey CA (2007). Health literacy and transplant patients and practitioners. Public Health.

[11] Gordon EJ, Wolf MS (2009). Health literacy skills of kidney transplant recipients. Prog Transplant.

[12] Kleinpeter MA (2003). Health literacy affects peritoneal dialysis performance and outcomes. Adv Perit Dial.

[13] Robinson JK, Guevara Y, Gaber R, Clayman ML, Kwasny MJ, Friedewald JJ, Gordon EJ (2014). Efficacy of a sun protection workbook for kidney transplant recipients: A randomized controlled trial of a culturally sensitive educational intervention. Am J Transplant.

[14] Baker DW, Williams MV, Parker RM, Gazmararian JA, Nurss J (1999). Development of a brief test to measure functional health literacy. Patient Educ Couns.

[15] Robinson JK, Turrisi R, Mallett KA, Stapleton J, Boone SL, Kim N, Riyat NV, Gordon EJ (2011). Efficacy of an educational intervention with kidney transplant recipients to promote skin self-examination for squamous cell carcinoma detection. Arch Dermatol.

[16] Katz MG, Jacobson TA, Veledar E, Kripalani S (2007). Patient literacy and question-asking behavior during the medical encounter: A mixed-methods analysis. J Gen Intern Med.

[17] Armstrong AW, Idriss NZ, Kim RH (2011). Effects of video-based, online education on behavioral and knowledge outcomes in sunscreen use: A randomized controlled trial. Patient Educ Couns.

[18] Tuong W, Larsen ER, Armstrong AW (2014). Videos to influence: A systematic review of effectiveness of video-based education in modifying health behaviors. J Behav Med.

